# Gene Signatures and Associated Transcription Factors of Allergic Rhinitis: *KLF4* Expression Is Associated with Immune Response

**DOI:** 10.1155/2023/1317998

**Published:** 2023-05-10

**Authors:** Youngsic Jeon, Tae Kyeom Kang, Wook-Bin Lee, Sang Hoon Jung, Young-Joo Kim

**Affiliations:** ^1^Natural Product Research Center, Korea Institute of Science and Technology, Gangneung, Republic of Korea; ^2^Division of Bio-Medical Science & Technology, KIST School, Korea University of Science and Technology, Gangneung, Republic of Korea

## Abstract

This study is aimed at investigating the potential molecular features of allergic rhinitis (AR) and identifying gene signatures and related transcription factors using transcriptome analysis and in silico datasets. Transcriptome profiles were obtained using three independent cohorts (GSE101720, GSE19190, and GSE46171) comprising healthy controls (HC) and patients with AR. The pooled dataset (*n* = 82) was used to identify the critical signatures of AR compared with HC. Subsequently, key transcription factors were identified by a combined analysis using transcriptome and in silico datasets. Gene ontology: bioprocess (GO: BP) analysis using differentially expressed genes (DEGs) revealed that immune response-related genes were significantly enriched in AR compared with HC. Among them, *IL1RL1*, *CD274*, and *CD44* were significantly higher in AR patients. We also identified key transcription factors between HC and AR using the in silico dataset and found that AR samples frequently express KLF transcription factor 4 (*KLF4*), which regulates immune response-related genes including *IL1RL1*, *CD274*, and *CD44* in human nasal epithelial cells. Our integrative analysis of transcriptomic regulation provides new insights into AR, which may help in developing precision management for patients with AR.

## 1. Introduction

Rhinitis is a highly prevalent multifactorial disease with heterogeneous conditions, consisting of allergic rhinitis (AR), nonallergic rhinitis, and infectious rhinitis. AR is characterized by inflammation of the nasal mucosa [1]; however, this classification may be oversimplified since many patients experience mixed symptoms and phenotypes [2, 3]. The diagnosis of AR has been evaluated using allergic symptoms (e.g., sneezing, coughing, itching, runny nose, and need to blow nose) and tests including skin prick and serum immunoglobulin E levels [4, 5]. However, these evaluations can be complex and inaccurate, necessitating the identification of better biomarkers to classify patients with AR and to develop targeted therapies.

To date, next-generation sequencing and bioinformatics analysis have been used to identify genetic and transcriptomic alterations involved in the development and progression of the disease and to classify patients for therapeutic prediction [6, 7]. Previous studies on AR have focused on genetic and transcriptomic alterations to identify AR-related features and have demonstrated variants of Vascular Endothelial Growth Factor B (*VEGFB*, 322A>C) and/or Integrin Subunit Alpha 2 (*ITGA2*, 502+1G>A) mainly in AR patients [8]. In addition, it was found that expression of the periostin (*POSTN*) gene was enhanced in AR compared to non-AR [9]. However, the molecular features of AR remain unclear; further research is needed to elucidate the key regulator of AR.

In this study, to determine the AR-related molecular signatures and genes, we performed transcriptome analysis of a pooled dataset consisting of 43 healthy controls (HC) and 39 AR samples. Our analysis exhibited an altered expression pattern of immune response genes in AR. In addition, using consensus clustering, we identified two AR subtypes (AR1 and AR2) and identified KLF transcription factor 4 (*KFL4*) as a potential candidate associated with immune- and cytokine-related AR subtypes. *KLF4* can act as a transcription factor regulating the expression of various target genes and is involved in the development of several epithelial tissues, such as skin and lungs [10–12]. Furthermore, *KLF4* is a critical regulator of monocyte differentiation [13]. Here, we demonstrated that interleukin-1*β* (IL-1*β*, encoded by *IL1B*), a proinflammatory cytokine induced by monocyte and dendritic cells, can enhance *KLF4* expression, resulting in upregulation of immune-related genes such as *IL1RL1*, *CD274*, and *CD44*. Our findings provide new insights into potential biomarkers for AR diagnosis and potential targeted therapy.

## 2. Materials and Methods

### 2.1. Transcriptome Data Analysis

Public datasets GSE101720, GSE19190, and GSE46171 were obtained from the NCBI Gene Expression Omnibus database (https://www.ncbi.nlm.nih.gov/geo/). Raw data were preprocessed by log_2_ transformation and quantile normalization, and the batch effects were corrected using empirical Bayes methods implemented in a sva R package “Combat” [14]. All data analyses were conducted in the RStudio environment (version 4.1.0). Samples with asthmatic tissues were excluded from the datasets (Supplementary Table 1). HC and AR consisted of the nasal mucosa and nasal airway epithelium.

Gene set analyses were performed using the gProfileR (0.7.0) package in R software and data from gene ontology: biological processes (GO: BP, http://geneontology.org/) and REAC (http://www.reactome.org/) databases. Coordinated gene regulation was identified using GSEA (https://www.gsea-msigdb.org/gsea, version 3.0) [15]. A genetic network was constructed using the GeneMANIA software in Cytoscape (version 3.4.1) [16].

### 2.2. Prediction of Transcription Factor Using *In Silico* Databases

Transcription factors were predicted using ChIP-X Enrichment Analysis version 3 (ChEA3, https://maayanlab.cloud/chea3/). ChEA3 was performed as previously described [17]. Transcription factor binding sites were analyzed by constructing matrices of immune-related genes analyzed using AliBaba2.1 (http://gene-regulation.com/pub/programs/alibaba2/index.html) [18].

### 2.3. Cell Culture and Cytokine Treatment

The human nasal epithelial cell line (HNEpC) purchased from Promocell (Heidelberg, Germany) was mainly grown in DetachKit (Promocell) at 37°C in a humidified atmosphere with 5% CO_2_. The cells were treated with inflammatory cytokines such as IL-1*β* for 24 h.

### 2.4. Cloning and Real-Time PCR

For the cloning of tagged *KLF4* coding gene sequences into pcDNA3.1 (-) (Addgene, Cambridge, MA, USA), the *KLF4* gene was amplified using total RNA extracted from the HEK293 cell line. PCR was performed using specific primers containing a 5′-extension and XbaI (NEB, Ipswich, MA, USA) and NotI (NEB) restriction sites with CloneAmp HiFi PCR Premix (Thermo Fisher Scientific, San Jose, CA, USA). Subsequently, the amplicons were digested with XbaI and NotI and cloned into the pcDNA3.1 (-) vector using an In-Fusion® cloning system according to the manufacturer's recommendations.

HNEpC cells were transfected with pcDNA3.1 (-) containing the tagged *KLF4* coding sequence using Lipofectamine® 3000 (Invitrogen, Carlsbad, CA, USA) according to the manufacturer's recommendations. Overexpression effects were validated by quantitative real-time PCR analysis using the iQ™ SYBR Supermix (Bio-Rad, CA, USA). The primers used in this study are summarized in Supplementary Table 2.

### 2.5. Statistical Analysis

Statistical analysis was performed using R software (version 3.4.0; Vienna, Austria). Statistical analyses were performed using Student's *t*-test, permutation *t*-test, or one-way ANOVA test.

## 3. Results

### 3.1. Differentially Expressed Genes between HC and AR

**Figure 1:**
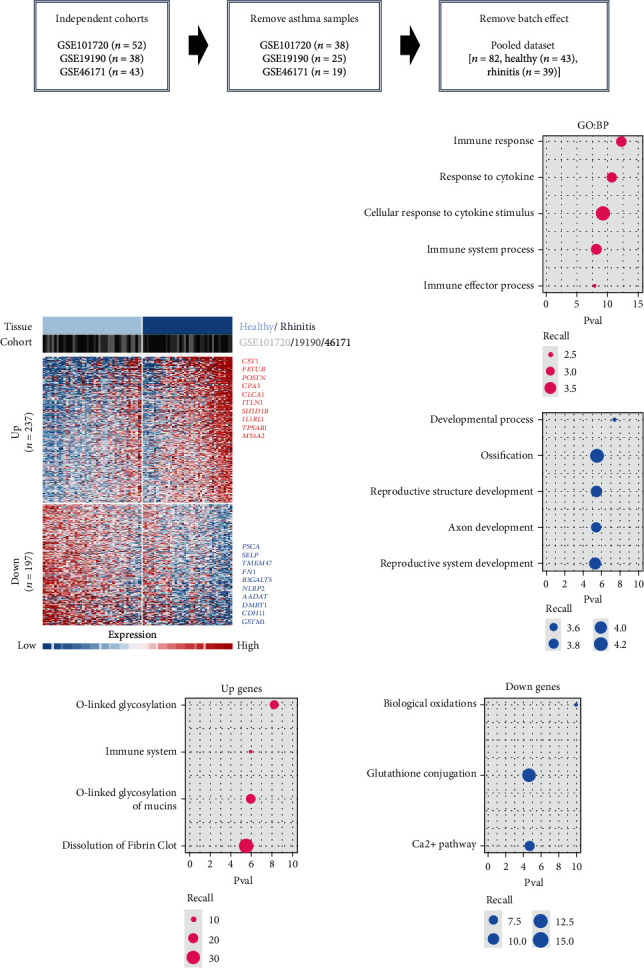
Differentially expressed genes between HC and AR. (a) A workflow showing the data parsing methods for transcriptome analysis. Pooled dataset is established (*n* = 82). (b) Heatmap showing the expression pattern of DEGs (up, *n* = 197; down, *n* = 237). The top genes are indicated on the right side. Different cohort information is shown (top). (c, d) The gene signatures enriched in “up” and “down” genes are shown using GO-BP and REAC databases. Plots represent the log_10_*P* value rate range detected across gProfileR (ver.0.7.0) levels for top-ranked signatures using pink color for up genes and blue color for down genes.

To acquire robust results with an extended HR and AR sample size, we first selected the independent transcriptome cohorts (e.g., GSE101720, GSE19190, and GSE46171), including 43 cases of HC and 39 cases of AR only composed of the nasal mucosa and nasal airway epithelium using GEO (*n* = 82). Next, we established the pooled transcriptome data using GEO datasets (Figure 1(a)). Then, we analyzed the differentially expressed genes (DEGs) between the HC and AR samples and classified them as rhinitis signatures “up” (*n* = 237) or “down” (*n* = 197) (*P* < 0.05, permutation *t*-test with a fold change (FC) > 0.3; Figure 1(b) and Supplementary Table 3), depending on whether they were upregulated or downregulated genes, respectively. Additionally, we sought to identify the underlying key signatures that potentially drive the phenotype difference between the groups. We then evaluated DEG-related signatures using GO: BP (*P* < 0.01, maximum *P* value with a minimum set size > 10). Gene set enrichment analysis revealed the enriched expression of immune- and cytokine-related genes in AR. We found that the tissue development-related signatures were frequently contained in the “down” genes (Figure 1(c)). Next, we validated the signatures of the DEGs. By applying the REAC database, we found that immune system-related genes were enriched in AR. In contrast, biological oxidation, glutathione conjugation, and Ca^2+^ pathway-related genes were enriched in HC (Figure 1(d)). Considering these results, we suggest that the immune system plays a pivotal role in AR pathogenesis.

### 3.2. Immune Response-Related Genes Are Enriched in AR

**Figure 2:**
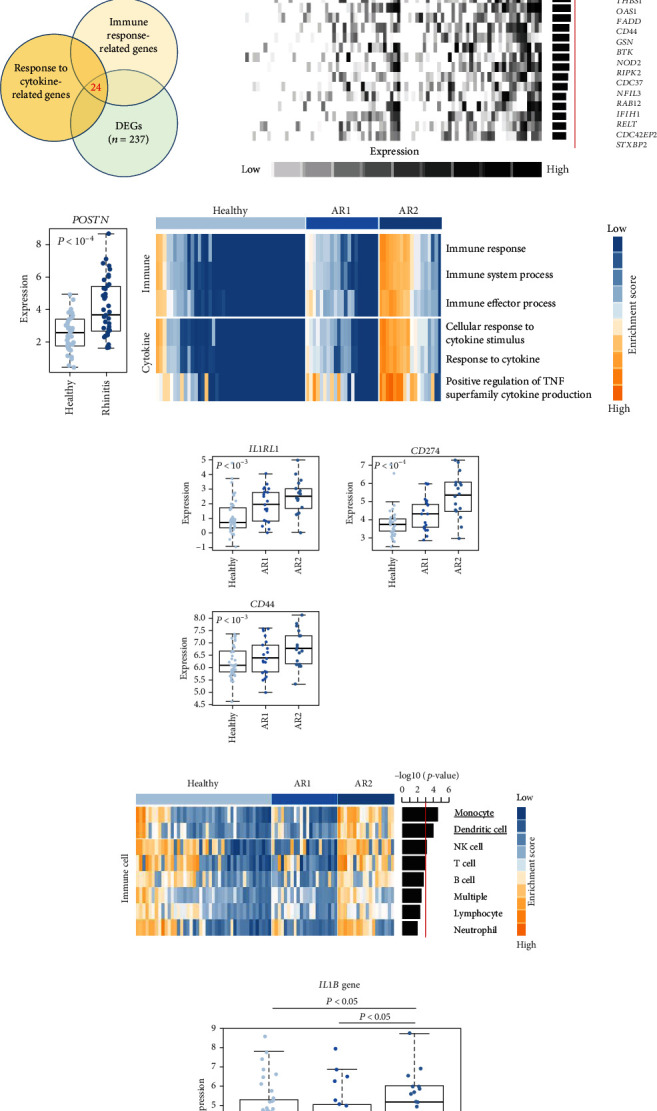
Immune response-related genes are enriched in AR. (a) A Venn diagram shows the overlapping genes among the immune response- and response to cytokine-related genes and DEGs between AR and HC. (b) Point plot showing the enrichment score of immune response (top). Heatmap showing the expression pattern of DEGs-dependent immune response-related genes (*n* = 54, bottom). A barplot showing the fold change of DEGs-dependent immune response-related genes (right). (c) Boxplots show the expression levels of *POSTN* (*P* value, Student *t*-test). (d) Heatmap shows the enrichment score of immune- and cytokine-related signatures. (e) Boxplots show the expression levels of *IL1RL1*, *CD274*, and *CD44* (*P* value, one-way ANOVA test). (f) Heatmap shows the enrichment score of immune cell-related signatures. (g) Boxplots show the expression levels of *IL1B* (*P* value, one-way ANOVA test).

Next, we focused on the underlying mechanisms involved in the altered expression of immune- and cytokine-related genes. Many immune-related genes were “response to cytokine”-dependent genes. We then sought to identify downstream players that drive the immune response with response to cytokines which had a significant correlation with the identified immune response signature and DEGs (*n* = 24, *P* < 0.05, permutation *t*-test with FC > 0.3, Figure 2(a)) and found that the enrichment score (ES) of the identified genes was significantly increased for AR compared with HC (*P* < 0.05, Figure 2(b), top). Among the immune-related DEGs, *IL1RL1* (FC = 1.12), *CD274* (FC = 0.90), and *CD44* (FC = 0.40) were the most significantly altered genes between the HC and AR groups (Figure 2(b), bottom). In addition, we evaluated the expression of a previously known biomarker of AR, *POSTN* [9]. The AR group exhibited high expression of *POSTN* compared with the HC group (Figure 2(c)).

As the AR samples showed different expression patterns of immune response genes, we performed consensus clustering (median absolute deviation > 0.7, *n* = 2,804). We analyzed the proportion of ambiguous clustering (PAC) score using the CDF curve and found that the optimal *K* was 2 with the lowest PAC value (Supplementary Figure 1(a)). Principal component analysis also revealed that AR samples were distributed in two separated fields (Supplementary Figure 1(b)). Based on these results, we classified AR into two robust subtypes, AR1 (*n* = 21) and AR2 (*n* = 18). We found that most of the immune- and cytokine-related signatures were significantly altered between AR1 and AR2 (Figure 2(d)), revealing a gradual increase in the expression of top genes and rhinitis marker genes (e.g., *IL1RL1*, *CD274*, and *CD44*) from healthy controls to AR1 and then AR2 (Figure 2(e)) [19, 20]. When we calculated the enrichment score (ES) for each group on the differentially expressed genes of immune cell-related signatures (Supplementary Table 4), we observed significantly higher ES of monocyte and dendritic cells in AR2 samples than other groups (*P* < 0.001, one-way ANOVA test, Figure 2(f)). Indeed, monocyte and dendritic cells are known to associate with allergic rhinitis and asthma [21, 22]. Moreover, we found that *IL1B* gene expression, a proinflammatory cytokine induced by monocyte and dendritic cells [23], was enhanced in AR2 compared to other groups (Figure 2(g)). These results indicate that AR samples show heterogeneous molecular subtypes, including AR1 and AR2.

### 3.3. Profiling of Transcription Factors Associated with Immune Response on HC and AR

**Figure 3:**
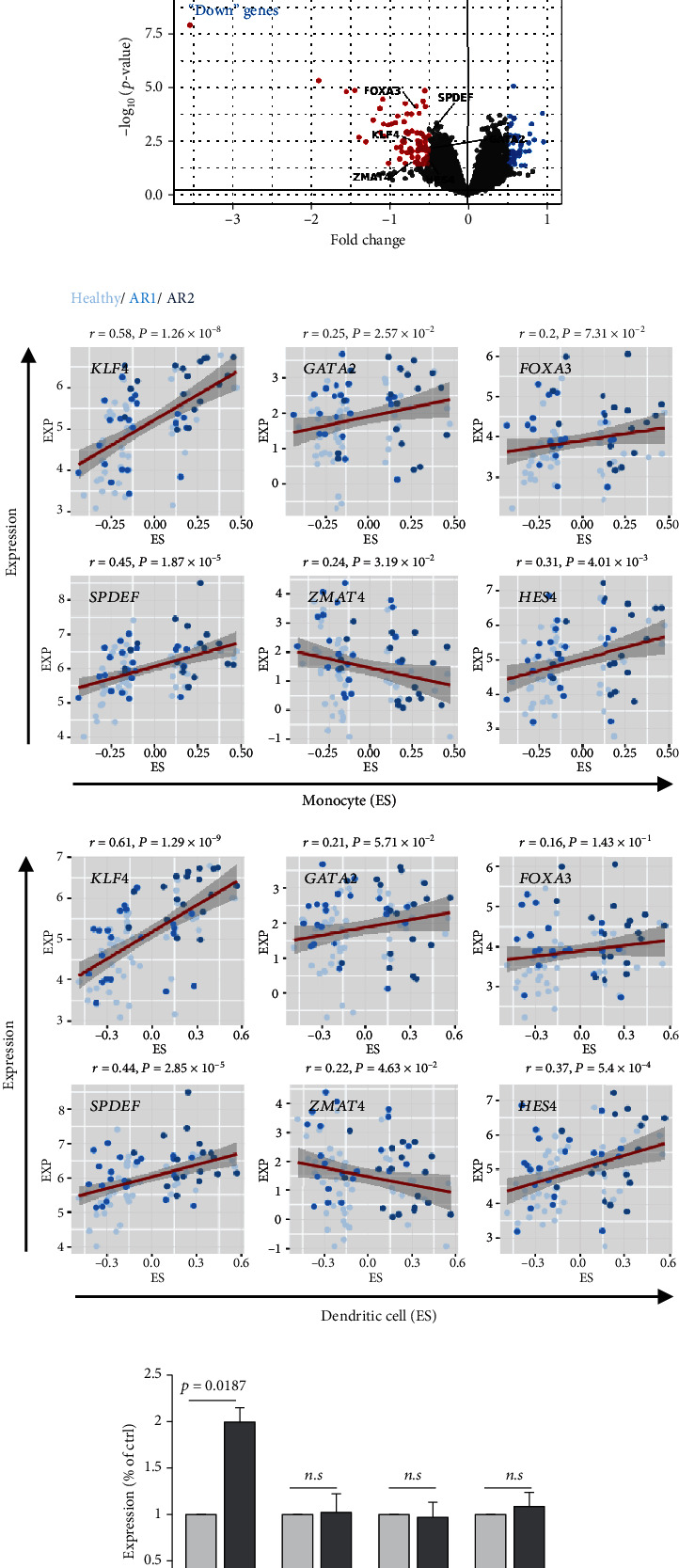
Profiling of transcription factors associated with immune response on HC and AR. (a) Heatmap shows the overlapping DEGs-dependent immune-related genes (*n* = 54) among top library (*n* = 51) by performing ChEA3 (https://maayanlab.cloud/chea3/). (b) Barplot shows the frequency of DEGs-dependent immune-related genes binding to predicted transcription factors (*n* = 1,632). The top 20 genes are indicated. (c) The volcano plot shows the distribution of DEGs. The DEGs-dependent transcription factors are indicated using black arrow. (d) Pairwise correlations for the expression levels of *GATA2*, *KLF4*, *FOXA3*, *SPDEF*, *ZMAT4*, and *HES4* and enrichment score of monocyte (up) and dendritic cell-related genes (bottom), respectively. (e) Barplot shows the expression levels of *KLF4*, *GATA2*, *SPDEF*, and *HES* in Ctrl and IL-1*β*-treated HNEpC cells. The expression levels of each gene were normalized to the expression level of *ACTB.* The data were presented as the mean ± SD values (*P* value, Student's *t*-test). (f, g) GSEA result showing the enriched expression of NF-*κ*B and STAT3-related genes.

As the transcription factor (TF) plays potential driver roles in immune response, we investigated the underlying TFs and the putative regulatory elements of immune-related genes. We identified 1,632 TFs predicted to bind to the promoter region of immune response-related genes (*n* = 54) using ChEA3 (see, for details, Materials and Methods, Figures 3(a) and 3(b)). Of these, we found that *KLF4*, *GATA2*, *FOXA3*, *SPDEF*, *ZMAT4*, and *HES4* were significantly altered between the HC and AR groups (*P* < 0.05, FC > 0.5, Figure 3(c)). Among them, *KLF4* showed the highest correlation value with immune-related genes compared to other transcription factors (*r* = 0.53, *P* < 3.27 × 10^−7^, Supplementary Figure 2). Furthermore, we observed that *KLF4* is significantly associated with the ES of monocyte- and dendritic cell-related genes compared to other transcription factors, respectively (*r* > 0.5, *P* < 10^−7^, Figure 3(d)).

The previous results demonstrated that AR2 was related to immune response, reflecting high expression of *IL1B*, a proinflammatory cytokine induced by monocyte and dendritic cells (see Figure 2). Thus, we evaluated whether *IL1B* is associated with *KLF4* expression. Next, we confirmed these findings via cell culture experiments, wherein treatment of IL-1*β* into a human nasal epithelial cell line significantly enhanced *KLF4* expression (*P* = 0.0187, Figure 3(e)). In contrast, other transcription factors such as *GATA2*, *SPDEF*, and *HES4* showed no effects. We also verified that gene set enrichment analysis revealed an enriched expression of NF-*κ*B and STAT3-related genes in the AR2, *KLF4* expression high group, compared to the HC and AR2 groups, respectively (Figures 3(f) and 3(g)). Indeed, IL-1*β* in immune response is known to induce the activation of the NF-*κ*B and STAT3 pathway [24, 25]. These findings strongly indicated that *KLF4* expression was associated with immune response via *IL1B* expression.

### 3.4. *KLF4* Transcription Factor Is Associated with the Immune Signature of AR

**Figure 4:**
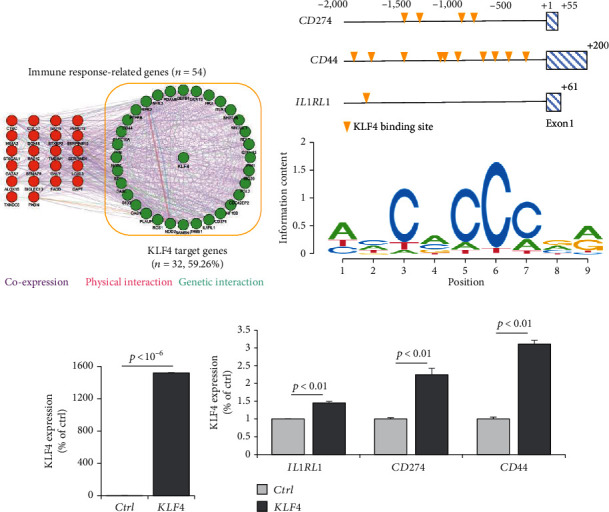
KLF4 transcription factor is associated with immune signature. (a) A genetic network of DEGs-dependent immune response-related genes was constructed showing coexpression (purple), physical interactions (pink), and genetic interactions (green) by using GeneMANIA software in Cytoscape (version 3.9.1). The KLF4 target genes are indicated. (b) The KLF4 transcription factor binding sites of each gene (e.g., *CD274*, *CD44*, and *IL1RL1*) are shown, which are predicted to bind KLF4 by performing AliBaba2.1 (http://gene-regulation.com/pub/programs/alibaba2/index.html) (up). Logos for binding motifs of KLF4 with 9 base pairs (bottom). (c) Barplot shows the expression levels of *KLF4* in Ctrl and *KLF4*-overexpressing HNEpC cells. (d) Barplot shows the expression levels of *IL1RL1*, *CD274*, and *CD44* in Ctrl and *KLF4*-overexpressing HNEpC cells. The expression levels of each gene were normalized to the expression level of *ACTB.* The data were presented as the mean ± SD values (*P* value, Student's *t*-test).

We also constructed a genetic network of immune response-related genes with *KLF4* and found that *KLF4* had a highly significant interaction with immune response-related genes (*IL1RL1*, *CD274*, *CD44*, etc., 32/54, 59.26%, Figure 4(a)). We also observed that KLF4 binds to the *CD274*, *CD44*, and *IL1RL1* gene promoter motifs (-2,000 to +100 bp from the transcription start site) (Figure 4(b), top). KLF4 binding domains showed sequences containing core 5′-CACCC-3′ elements (Figure 4(b), bottom). In addition, *KLF4* expression gradually increased from HC to AR1 and then AR2 (*P* < 0.01, Supplementary Figure 3).

To verify whether *KLF4* expression can affect the immune response-related genes, we established a *KLF4* overexpression system in a human nasal epithelial cell line (HNEpC) that has lower expression levels of *KLF4* (*P* < 10^−6^, Figure 4(c)). *KLF4* overexpression cells could induce *IL1RL1*, *CD274*, and *CD44* genes (*P* < 10^−6^, Figure 4(d)). Thus, it is plausible that *KLF4* expression enhances immune response-related genes, resulting in immune response-related signatures.

## 4. Discussion

Several studies have been conducted to identify candidate genes associated with rhinitis. However, these studies were conducted with limited sample numbers and require further evaluation [9, 26]. As such, the molecular mechanisms underlying AR remain unclear.

In this study, we established a pooled transcriptome data using three independent cohorts (summarized in Figure 1(a)) and demonstrated a difference in transcriptome signatures between the HC and AR samples. Among the identified signatures, AR was significantly enriched in the immune response and response to cytokine signatures compared with HC. In addition, using consensus clustering, we identified two AR subtypes (AR1 and AR2) and found that immune- and cytokine-related signatures were significantly lower in the AR1 subtype. In contrast, AR2 showed a high expression of the immune-related genes (e.g., *IL1RL1*, *CD274*, and *CD44*) which are associated with the *KLF4* transcription factor using transcriptome analysis based on an in silico database.

As the epithelium of the AR is a region where the population of allergen-presenting cells is concentrated, the AR immune system has been associated with the expression of cytokines [27]. AR indicated a high expression of immune- and cytokine-related genes, including *IL1RL1*, *CD274*, and *CD44*, compared with HC. These genes have been associated with well-known rhinitis marker genes [20, 28]. A recent study demonstrated that genetic variations in *IL1RL1* are strongly associated with asthma, revealing that *IL1RL1* acts as an essential driver of type 2 immune responses to IL-33 [29]. Moreover, *IL1RL1* is a reliable marker of Th2 lymphocytes in AR, and *IL1RL1* variants (e.g., rs72823628, rs950881, and rs3771175) are associated with AR risk [19, 30, 31]. However, in this study, the association of these variants and others between AR1 and AR2 was not identified, which is a limitation of this study and should be confirmed in the near future. *CD274* and *CD44* have emerged as critical immune regulators associated with T cell receptor blockade and the development of airway inflammation [20, 32]. In this study, we showed that the AR transcriptome has heterogeneities, revealing two robust subtypes, AR1 and AR2, using consensus clustering. Also, we demonstrated that AR2 subtype has higher gene expression of *IL1RL1*, *CD274*, and *CD44* compared to AR1 and HC. This finding implies that AR is divided by immune traits, although further elucidation is required.

Via in silico analysis, we found that the *KLF4* transcription factor was significantly associated with immune response genes, and it was highly expressed in AR2. As *KLF4* is known to be an immune marker for dendritic cell differentiation and essential for inflammatory monocyte differentiation [33, 34], our results indicate that putative regulatory elements of immune response-related genes such as *IL1RL1*, *CD274*, and *CD44* are strongly related to *KLF4*. However, it should be noted that we did not evaluate the validity of our results using an independent rhinitis cohort, which requires further extended studies in the near future. In addition, the various multifaced functions of the *KLF4* have been known previously, including cell proliferation, differentiation, development, and transcription [10], which should be validated to understand more relevant mechanisms of immune-related gene expression in AR.

In conclusion, we suggest that the expression of *KLF4* is an independent predictor of AR. *KLF4* expression is associated with IL-1*β*, a proinflammatory cytokine induced by monocyte and dendritic cells, and may contribute to the expression of immune response-related gene transcription. Thus, targeting *KLF4* could be a promising therapeutic strategy for AR patients.

## Data Availability

The transcriptome data are available in GEO at https://www.ncbi.nlm.nih.gov/geo/, accession numbers GSE101720, GSE19190, and GSE46171. These data were derived from the following resources available in the public domain: https://www.ncbi.nlm.nih.gov/geo/query/acc.cgi?acc=GSE10720, https://www.ncbi.nlm.nih.gov/geo/query/acc.cgi?acc=GSE19190, and https://www.ncbi.nlm.nih.gov/geo/query/acc.cgi?acc=GSE46171, respectively.

## Supplementary Materials

Supplementary Materials Supplementary Figure 1: allergic rhinitis samples reveal two subtypes using consensus clustering. Supplementary Figure 2: expression of transcription factors is correlated with the enrichment score of immune response. Supplementary Figure 3: differences in expression of transcription factors across the subtypes. Supplementary Table 1: list of information used in rhinitis analysis. Supplementary Table 2: primers used for cloning and qRT-PCR. Supplementary Table 3: list of differentially expressed genes (DEGs) between the HC and AR samples. Supplementary Table 4: list of information used in immune cell signature.
