# Supplementary material for: Gene Signatures and Associated Transcription Factors of Allergic Rhinitis: KLF4 Expression Is Associated with Immune Response
Source: Biomed Res Int. 2023 May 10;2023:1317998. doi: 10.1155/2023/1317998 (PMC10191743; doi:10.1155/2023/1317998)
Supplement: Supplementary Materials — Supplementary Figure 1: allergic rhinitis samples reveal two subtypes using consensus clustering. Supplementary Figure 2: expression of transcription factors is correlated with the enrichment score of immune response. Supplementary Figure 3: differences in expression of transcription factors across the subtypes. Supplementary Table 1: list of information used in rhinitis analysis. Supplementary Table 2: primers used for cloning and qRT-PCR. Supplementary Table 3: list of differentially expressed genes (DEGs) between the HC and AR samples. Supplementary Table 4: list of information used in immune cell signature. [file 1317998.f1.docx]

**Supplementary Materials for:**

**Gene signatures and associated transcription factors of allergic rhinitis: *KLF4* expression is associated with immune response.**

Youngsic Jeon^1^, Tae Kyeom Kang^1^, Wook-Bin Lee^1^, Sang Hoon Jung^1^, Young-Joo Kim^1^

^1^ Natural Product Research Center, Korea Institute of Science and Technology, Gangneung, South Korea


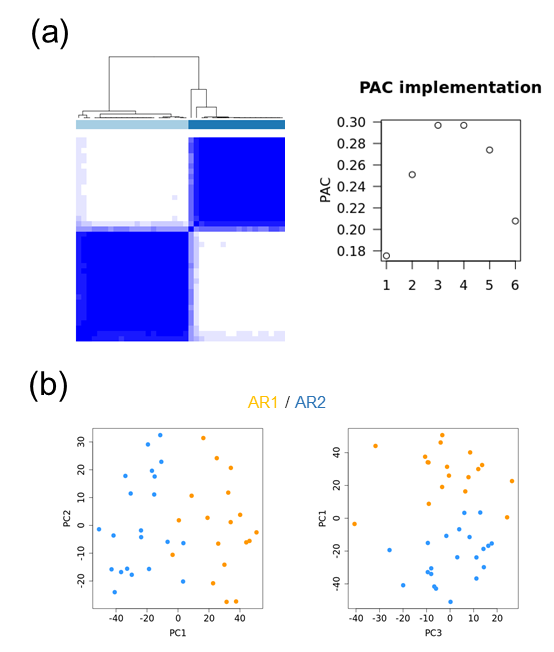


## Supplementary Figure 1. Allergic rhinitis samples reveal two subtypes using consensus clustering

(**a**) A heatmap shows unsupervised clustering analysis using variably expressed genes (median absolute deviation, MAD > 0.7, n = 6,399) (*left*). A plot shows the PAC (proportion of ambiguous clustering) values across the different K numbers (*right*). (**b**) Principal component analysis using the variable genes (MAD > 0.7, n = 6,399) shows that AR1 and AR2 samples are distributed in the two areas.


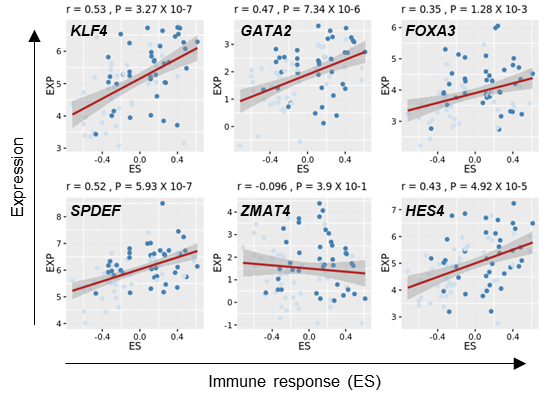


## Supplementary Figure 2. Expression of transcription factors is correlated with the enrichment score of immune response

Correlation for the expression levels of transcription factors (e.g., *KLF4*, *GATA2*, *FOXA3*, *SPDEF*, *ZMAT4*, and *HES4*) with enrichment score of immune response is shown, respectively.


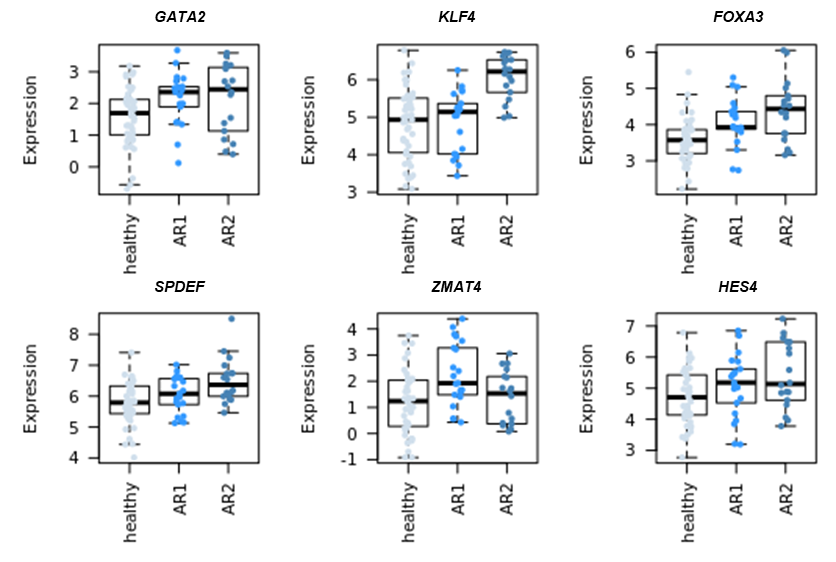


## Supplementary Figure 3. Differences in expression of transcription factors across the subtypes

Boxplots show the expression levels of the transcription factors (*i.e.*, *GATA2*, *KLF4*, *FOXA3*, *SPDEF*, *ZMAT4*, and *HES4*).

**Supplementary table 1. List of information used in rhinitis analysis**

| Sample | GSE101720 | GSE19190 | GSE46171 |
| --- | --- | --- | --- |
| healthy | 18 | 11 | 14 |
| rhinitis | 20 | 14 | 5 |
| asthma or rhinitis+asthma | 14 | 13 | 25 |

**Supplementary table 2. Primers used for cloning and qRT-PCR**

| **Primer** | **Purpose** | **Sequence (5’→3’)** | **Annealing** |
| --- | --- | --- | --- |
| *KLF4* F^a^ | qRT-PCR | ACCCTGGGTCTTGAGGAAGT | 62°C |
| *KLF4* R^b^ |  | GCAGGAAGGATGGGTAATTG |  |
| *GATA2* F | qRT-PCR | GTCACTGACGGAGAGCATGA | 58°C |
| *GATA2* R |  | GTCACTGACGGAGAGCATGA |  |
| *HES4* F | qRT-PCR | CTCACGGTCATCTCCAGGAT | 62°C |
| *HES4* R |  | CTCACGGTCATCTCCAGGAT |  |
| *SPDEF* F | qRT-PCR | GACCAGTGAGGAGAGCTGGA | 62°C |
| *SPDEF* R |  | CATAGCTGTGGGGCTTGAGT |  |
| *CD247 F* | qRT-PCR | GTCACTGACGGAGAGCATGA | 62°C |
| *CD274 R* |  | CAGGACTTGATGGTCACTGC |  |
| *CDC37 F* | qRT-PCR | GCCATGAAGGAGTACGAGGA | 58°C |
| *CDC37 R* |  | CACATCGAAGCACTTCTGGA |  |
| *IL1RL1 F* | qRT-PCR | TGAGGACGCAGGTGATTACA | 58°C |
| *IL1RL1 R* |  | AAAGCCTTGCTCATCCTTGA |  |
| *CD44* F | qRT-PCR | AAGGTGGAGCAAACACAACC | 62°C |
| *CD44* R |  | ACTGCAATGCAAACTGCAAG |  |
| *KLF4* *XbaI* F | Cloning | AAACGGGCCCTCTAGATGAGGCAGCCACCTGGC | 64°C |
| *KLF4 NotI* R |  | AGCACAGTGGCGGCCTTAAAAATGCCTCTTCATGTGTAAG |  |
| qRT-PCR Quantitative Reverse Transcription PCR  ^a^Forward  ^b^Reverse. | | | |

**Supplementary table 3. List of differentially expressed genes (DEGs) between the HC and AR samples**

|  | Up genes (n = 237) | | |  | Down genes (n = 197) | | |
| --- | --- | --- | --- | --- | --- | --- | --- |
| No. | Symbol | p-value^a^ | FC^b^ |  | Symbol | p-value | FC |
| 1 | *CST1* | 1.37E-08 | 3.5243977 |  | *GSTM1* | 3.70E-03 | -0.9500489 |
| 2 | *FETUB* | 5.26E-06 | 1.9043477 |  | *CDH11* | 1.63E-04 | -0.9383819 |
| 3 | *POSTN* | 1.71E-05 | 1.5594314 |  | *DMBT1* | 2.96E-03 | -0.8363459 |
| 4 | *CPA3* | 1.50E-05 | 1.4525171 |  | *AADAT* | 5.27E-04 | -0.8028738 |
| 5 | *CLCA1* | 2.23E-03 | 1.380447 |  | *NLRP2* | 1.55E-03 | -0.7783233 |
| 6 | *ITLN1* | 3.68E-03 | 1.2928318 |  | *B3GALT5* | 9.84E-03 | -0.7732053 |
| 7 | *SH2D1B* | 3.37E-04 | 1.2016773 |  | *FN1* | 4.10E-03 | -0.7661619 |
| 8 | *IL1RL1* | 9.75E-05 | 1.1181914 |  | *TMEM47* | 1.03E-02 | -0.7237705 |
| 9 | *TPSAB1* | 1.34E-03 | 1.1089322 |  | *SELP* | 4.59E-02 | -0.7138867 |
| 10 | *MS4A2* | 3.66E-05 | 1.0841627 |  | *PSCA* | 4.64E-02 | -0.6716114 |
| 11 | *ANO1* | 5.33E-04 | 1.0730112 |  | *SSPN* | 4.14E-03 | -0.6704445 |
| 12 | *PHLDB2* | 1.98E-03 | 1.0645536 |  | *USP6* | 5.40E-03 | -0.6639528 |
| 13 | *SERPINB2* | 5.86E-04 | 1.0131648 |  | *CCDC80* | 9.08E-03 | -0.6548309 |
| 14 | *CPA4* | 3.66E-02 | 1.0063966 |  | *SULT1E1* | 1.29E-02 | -0.6509908 |
| 15 | *GSDMC* | 5.35E-04 | 0.9706764 |  | *WFDC6* | 1.65E-02 | -0.6416363 |
| 16 | *SERPINB10* | 4.54E-04 | 0.9257936 |  | *MACROD2* | 2.04E-03 | -0.6346174 |
| 17 | *CISH* | 6.86E-03 | 0.9087659 |  | *CCDC3* | 1.24E-02 | -0.6271383 |
| 18 | *CD274* | 2.18E-04 | 0.8959599 |  | *BHLHE41* | 2.57E-03 | -0.6164554 |
| 19 | *SLC9A3* | 2.31E-02 | 0.8654806 |  | *MFAP3L* | 2.42E-03 | -0.607879 |
| 20 | *PTHLH* | 6.25E-03 | 0.8505879 |  | *HGD* | 1.86E-03 | -0.5978611 |
| 21 | *TMSB4Y* | 1.06E-02 | 0.8397151 |  | *GPX3* | 1.69E-02 | -0.5892029 |
| 22 | *SNTG2* | 3.49E-03 | 0.8286764 |  | *EDIL3* | 4.15E-02 | -0.588618 |
| 23 | *ADAMTS9* | 3.04E-03 | 0.8143899 |  | *COL12A1* | 2.72E-04 | -0.5842973 |
| 24 | *HPCAL1* | 4.04E-04 | 0.813928 |  | *DDIT4L* | 2.29E-02 | -0.5812449 |
| 25 | *DPP4* | 3.86E-03 | 0.8085637 |  | *ACSS3* | 2.91E-04 | -0.5800788 |
| 26 | *CMYA5* | 5.72E-05 | 0.8002128 |  | *F5* | 3.24E-02 | -0.5795783 |
| 27 | *TFF1* | 3.72E-02 | 0.795755 |  | *PAPPA* | 9.09E-06 | -0.5761496 |
| 28 | *DNAJC12* | 1.31E-03 | 0.7879058 |  | *TRIM36* | 1.49E-02 | -0.576117 |
| 29 | *PXDN* | 1.18E-02 | 0.7763014 |  | *AR* | 1.04E-02 | -0.5729192 |
| 30 | *PCSK6* | 1.74E-04 | 0.7742246 |  | *BSN* | 4.05E-03 | -0.568748 |
| 31 | *GCNT3* | 1.24E-03 | 0.7725787 |  | *SLC26A7* | 2.28E-02 | -0.5687128 |
| 32 | *PTGS1* | 1.48E-03 | 0.7635346 |  | *ECM2* | 1.02E-02 | -0.5659703 |
| 33 | *LRRC31* | 7.60E-03 | 0.7399681 |  | *CYB5A* | 2.39E-02 | -0.5649462 |
| 34 | *NTRK2* | 1.18E-02 | 0.7276967 |  | *MUC5B* | 1.36E-02 | -0.5525889 |
| 35 | *RTKN2* | 7.65E-03 | 0.7266767 |  | *CA8* | 5.03E-04 | -0.5499967 |
| 36 | *CDH26* | 1.76E-04 | 0.7249039 |  | *DSEL* | 2.41E-02 | -0.5473026 |
| 37 | *VAMP5* | 1.93E-02 | 0.7116662 |  | *SLIT2* | 1.01E-02 | -0.5422979 |
| 38 | *DOK1* | 1.14E-03 | 0.711643 |  | *SPOCK3* | 3.47E-02 | -0.5397553 |
| 39 | *OASL* | 1.00E-02 | 0.7086224 |  | *EGFR* | 2.23E-04 | -0.5361555 |
| 40 | *AKAP12* | 3.35E-02 | 0.669698 |  | *ANKH* | 1.81E-03 | -0.5318975 |
| 41 | *SLC17A5* | 7.56E-05 | 0.6622722 |  | *HLF* | 2.99E-02 | -0.5301373 |
| 42 | *PTPRH* | 3.17E-02 | 0.6572582 |  | *NFIB* | 6.44E-04 | -0.5292446 |
| 43 | *KDELR3* | 1.39E-03 | 0.6481445 |  | *CYP24A1* | 4.56E-02 | -0.5086747 |
| 44 | *B3GNT6* | 3.15E-02 | 0.6436653 |  | *SPARC* | 1.47E-02 | -0.5063141 |
| 45 | *SLC22A16* | 4.03E-02 | 0.6429176 |  | *GPLD1* | 4.83E-03 | -0.5015256 |
| 46 | *SYCP2* | 3.99E-02 | 0.6399808 |  | *SLC6A15* | 2.81E-02 | -0.5001009 |
| 47 | *KCNK6* | 3.29E-03 | 0.638886 |  | *NRCAM* | 9.60E-03 | -0.4994778 |
| 48 | *UPK1B* | 1.01E-02 | 0.6383516 |  | *ABCA4* | 2.56E-03 | -0.4966778 |
| 49 | *ZMAT4* | 1.93E-02 | 0.6369064 |  | *TRAM1L1* | 1.44E-02 | -0.4966764 |
| 50 | *CD69* | 4.47E-03 | 0.6314214 |  | *MYH10* | 2.15E-02 | -0.4943962 |
| 51 | *ISG20* | 8.03E-03 | 0.6300881 |  | *EPDR1* | 8.96E-04 | -0.4918667 |
| 52 | *FOXA3* | 1.62E-04 | 0.6270453 |  | *RBMS3* | 1.26E-02 | -0.4899437 |
| 53 | *HK3* | 4.17E-02 | 0.6217055 |  | *ARSD* | 2.83E-03 | -0.4873398 |
| 54 | *GNLY* | 8.78E-03 | 0.6135098 |  | *MAOB* | 1.95E-02 | -0.4824876 |
| 55 | *TFF3* | 3.74E-03 | 0.6104135 |  | *CSRNP3* | 2.23E-02 | -0.4805662 |
| 56 | *KLF4* | 5.22E-03 | 0.609017 |  | *GATM* | 9.79E-03 | -0.4791434 |
| 57 | *TTPA* | 5.99E-03 | 0.6060186 |  | *PCDHB11* | 8.35E-03 | -0.4767562 |
| 58 | *PYCR1* | 6.97E-03 | 0.6051415 |  | *LRP1B* | 5.36E-03 | -0.4753441 |
| 59 | *SERPINB4* | 1.42E-03 | 0.5998935 |  | *TOX* | 1.76E-03 | -0.4747392 |
| 60 | *CA2* | 4.16E-02 | 0.583867 |  | *ALDH3A1* | 2.43E-03 | -0.4719749 |
| 61 | *NOS2* | 3.94E-02 | 0.5836581 |  | *SLC13A2* | 3.00E-04 | -0.4711184 |
| 62 | *ST6GAL1* | 1.65E-03 | 0.5820628 |  | *DCLK1* | 1.25E-02 | -0.4710284 |
| 63 | *CEP72* | 4.51E-05 | 0.5763706 |  | *ESD* | 2.65E-02 | -0.4621131 |
| 64 | *SLC18A2* | 7.95E-03 | 0.5744694 |  | *LRIG3* | 6.87E-03 | -0.4619911 |
| 65 | *NTS* | 2.56E-02 | 0.5664582 |  | *TAS2R43* | 1.84E-02 | -0.4613728 |
| 66 | *GATA2* | 8.06E-03 | 0.5655516 |  | *FAM107A* | 3.12E-02 | -0.4574916 |
| 67 | *PDE10A* | 8.69E-03 | 0.5631247 |  | *SORT1* | 4.35E-04 | -0.4545655 |
| 68 | *FBN2* | 2.21E-03 | 0.559827 |  | *GSTA2* | 3.52E-02 | -0.4511498 |
| 69 | *SLC7A1* | 1.48E-05 | 0.5565885 |  | *CHL1* | 3.04E-02 | -0.4492148 |
| 70 | *ELOVL5* | 7.88E-05 | 0.5483965 |  | *BBS2* | 3.39E-02 | -0.4488686 |
| 71 | *SEMA7A* | 3.72E-03 | 0.5454195 |  | *FZD3* | 2.22E-02 | -0.4483575 |
| 72 | *CEACAM21* | 6.64E-03 | 0.5434925 |  | *CMBL* | 9.97E-03 | -0.4450633 |
| 73 | *SERPINE2* | 2.65E-02 | 0.5425366 |  | *SVOPL* | 3.00E-03 | -0.4447476 |
| 74 | *ISG15* | 2.22E-02 | 0.5424183 |  | *TMEM37* | 7.49E-03 | -0.4420698 |
| 75 | *GCNT4* | 5.79E-03 | 0.5403395 |  | *GRIK2* | 3.62E-03 | -0.4418711 |
| 76 | *HAS3* | 9.51E-03 | 0.5388885 |  | *AKR1C3* | 2.83E-03 | -0.4368828 |
| 77 | *HBEGF* | 2.28E-03 | 0.5364387 |  | *LEF1* | 2.58E-03 | -0.4368445 |
| 78 | *PTPRB* | 2.28E-02 | 0.5185612 |  | *EPHX1* | 4.82E-03 | -0.4317359 |
| 79 | *HES4* | 1.77E-02 | 0.5180166 |  | *SLC16A12* | 2.49E-02 | -0.4281189 |
| 80 | *MLKL* | 8.22E-03 | 0.5151594 |  | *CDCA7* | 4.82E-03 | -0.4279654 |
| 81 | *BCAT1* | 3.92E-02 | 0.5133237 |  | *BCAM* | 1.81E-02 | -0.4277663 |
| 82 | *KRT23* | 7.97E-03 | 0.505331 |  | *C3* | 4.47E-02 | -0.4268985 |
| 83 | *BCL2L15* | 1.01E-02 | 0.5036397 |  | *PDE5A* | 3.69E-03 | -0.4226957 |
| 84 | *SPDEF* | 1.87E-03 | 0.5000001 |  | *RGS5* | 3.21E-02 | -0.4206381 |
| 85 | *TRIM47* | 9.46E-04 | 0.4986281 |  | *NUDT4* | 2.91E-03 | -0.4205819 |
| 86 | *FGFBP1* | 3.66E-02 | 0.4974672 |  | *SCPEP1* | 7.35E-03 | -0.4178363 |
| 87 | *DGKI* | 3.89E-02 | 0.495952 |  | *PDE7B* | 8.57E-03 | -0.41743 |
| 88 | *GPR183* | 1.97E-02 | 0.4924682 |  | *ITPR1* | 2.22E-02 | -0.4163599 |
| 89 | *NRXN3* | 3.33E-02 | 0.4860687 |  | *SNRNP200* | 4.67E-04 | -0.412201 |
| 90 | *GJB5* | 2.06E-02 | 0.4810763 |  | *SGCB* | 9.07E-03 | -0.4092833 |
| 91 | *LBH* | 2.90E-03 | 0.4795014 |  | *GLI3* | 4.99E-03 | -0.4087004 |
| 92 | *FUT3* | 1.13E-02 | 0.4750708 |  | *HEY2* | 4.40E-02 | -0.4080955 |
| 93 | *CSTA* | 1.87E-02 | 0.4716432 |  | *A2M* | 1.02E-02 | -0.4060391 |
| 94 | *EPHA4* | 2.82E-03 | 0.4651517 |  | *ATP6V0A4* | 3.78E-03 | -0.4054749 |
| 95 | *HS3ST1* | 7.17E-03 | 0.4641809 |  | *PRPF8* | 4.83E-04 | -0.4054073 |
| 96 | *PLAUR* | 4.54E-02 | 0.4633425 |  | *HSPBAP1* | 2.88E-03 | -0.4052192 |
| 97 | *SLC6A14* | 8.52E-03 | 0.4591424 |  | *DHCR24* | 3.24E-03 | -0.4043743 |
| 98 | *MYO3A* | 4.14E-02 | 0.4564592 |  | *CACHD1* | 2.00E-04 | -0.4029239 |
| 99 | *MTHFD2* | 1.16E-02 | 0.4558698 |  | *CACNA2D3* | 1.77E-02 | -0.4025273 |
| 100 | *CTSC* | 3.21E-03 | 0.4538076 |  | *TNFSF4* | 3.02E-02 | -0.4016138 |
| 101 | *IL3RA* | 1.89E-02 | 0.4521183 |  | *WDR17* | 3.24E-02 | -0.400543 |
| 102 | *SERPINB13* | 3.83E-03 | 0.4513481 |  | *GPM6B* | 4.85E-02 | -0.3995541 |
| 103 | *PROCR* | 3.31E-02 | 0.4490491 |  | *PBX1* | 1.00E-02 | -0.3986985 |
| 104 | *DQX1* | 1.21E-03 | 0.4488818 |  | *OVGP1* | 8.95E-03 | -0.3967771 |
| 105 | *PRTFDC1* | 3.21E-02 | 0.4460908 |  | *TMEM14A* | 8.96E-03 | -0.3948723 |
| 106 | *LOXL3* | 1.28E-02 | 0.4447364 |  | *ALDH5A1* | 1.83E-03 | -0.3944723 |
| 107 | *DSE* | 6.57E-03 | 0.4436921 |  | *APCDD1* | 4.86E-02 | -0.3937351 |
| 108 | *DDX58* | 1.52E-02 | 0.4414762 |  | *LRP1* | 1.55E-03 | -0.3891169 |
| 109 | *ZNF467* | 1.31E-02 | 0.4410919 |  | *TET1* | 1.15E-02 | -0.3882814 |
| 110 | *ATP12A* | 5.55E-03 | 0.4409523 |  | *ACOX2* | 1.43E-02 | -0.3880983 |
| 111 | *RGS7BP* | 2.54E-02 | 0.437878 |  | *HTRA1* | 9.11E-03 | -0.3858664 |
| 112 | *DEFB1* | 4.84E-02 | 0.4368958 |  | *FBN1* | 1.62E-02 | -0.3852763 |
| 113 | *SERPINB8* | 1.81E-02 | 0.4352487 |  | *DTX4* | 1.91E-03 | -0.3851641 |
| 114 | *SAMSN1* | 2.79E-02 | 0.4347327 |  | *TTC28* | 3.96E-03 | -0.3811428 |
| 115 | *HDC* | 2.20E-02 | 0.4343092 |  | *ANK3* | 1.70E-03 | -0.3805143 |
| 116 | *PPM1K* | 2.84E-03 | 0.4339308 |  | *MMP2* | 2.01E-02 | -0.3795862 |
| 117 | *GALE* | 2.56E-03 | 0.4322531 |  | *PGBD1* | 4.08E-02 | -0.3792885 |
| 118 | *ALOX15* | 1.55E-03 | 0.4246949 |  | *ARSG* | 1.08E-02 | -0.3788782 |
| 119 | *THBS1* | 7.19E-03 | 0.4191949 |  | *KBTBD11* | 3.78E-02 | -0.3761874 |
| 120 | *XKR6* | 2.59E-02 | 0.4171788 |  | *TUT1* | 1.15E-03 | -0.3753184 |
| 121 | *GAPT* | 1.15E-02 | 0.4129418 |  | *WNT5A* | 3.36E-03 | -0.3750015 |
| 122 | *OAS1* | 4.29E-02 | 0.4104435 |  | *ZRANB3* | 3.60E-03 | -0.374796 |
| 123 | *HK2* | 1.60E-02 | 0.4095335 |  | *CABYR* | 3.82E-02 | -0.3743348 |
| 124 | *CACNB4* | 3.74E-02 | 0.4075293 |  | *RPS6KA6* | 1.46E-02 | -0.3734877 |
| 125 | *P2RY6* | 3.84E-02 | 0.4049194 |  | *ATP2B4* | 1.36E-03 | -0.3731893 |
| 126 | *FADD* | 3.72E-03 | 0.4046286 |  | *HEY1* | 1.99E-02 | -0.3692011 |
| 127 | *ST3GAL4* | 8.80E-03 | 0.4028993 |  | *SNTB1* | 4.93E-02 | -0.3683753 |
| 128 | *UGCG* | 5.00E-04 | 0.4025112 |  | *AHNAK* | 2.60E-03 | -0.3679268 |
| 129 | *SHC2* | 1.22E-02 | 0.4012657 |  | *GCLC* | 6.26E-03 | -0.3678877 |
| 130 | *PMM2* | 8.58E-03 | 0.3999881 |  | *POLR1B* | 1.36E-02 | -0.3676162 |
| 131 | *HRH1* | 9.29E-03 | 0.3979326 |  | *PLCB2* | 1.04E-02 | -0.3676066 |
| 132 | *ATG16L1* | 6.61E-04 | 0.3973553 |  | *ATL1* | 1.47E-02 | -0.3651423 |
| 133 | *LRRC8D* | 5.44E-03 | 0.3952386 |  | *DTD1* | 2.16E-03 | -0.3638121 |
| 134 | *ADAMTSL3* | 1.18E-02 | 0.3931814 |  | *SLC23A2* | 7.07E-03 | -0.3624638 |
| 135 | *VWF* | 1.83E-02 | 0.3879962 |  | *MTR* | 5.96E-04 | -0.3589131 |
| 136 | *GALNT5* | 3.55E-02 | 0.3873543 |  | *RPA1* | 4.56E-03 | -0.3587285 |
| 137 | *FAM50A* | 4.64E-03 | 0.3872424 |  | *UBE2H* | 6.45E-03 | -0.3557957 |
| 138 | *CLEC10A* | 2.49E-02 | 0.3871739 |  | *AKR1C1* | 1.71E-02 | -0.3542213 |
| 139 | *AKAP5* | 2.68E-02 | 0.3870655 |  | *POR* | 2.18E-03 | -0.3498586 |
| 140 | *DNAJC1* | 9.36E-04 | 0.384597 |  | *PEBP1* | 5.22E-03 | -0.346946 |
| 141 | *VPS37B* | 1.15E-02 | 0.3836759 |  | *DUSP4* | 1.03E-02 | -0.3455709 |
| 142 | *CD44* | 1.41E-02 | 0.3816448 |  | *SHMT1* | 3.06E-03 | -0.3454947 |
| 143 | *RNF19B* | 5.24E-03 | 0.3814734 |  | *MAGEE1* | 1.16E-02 | -0.3446662 |
| 144 | *SH2D3C* | 1.56E-02 | 0.3812169 |  | *BCL2L11* | 4.66E-02 | -0.3443395 |
| 145 | *GSN* | 6.68E-04 | 0.3806288 |  | *ENAH* | 2.33E-02 | -0.3443283 |
| 146 | *ARNTL2* | 2.89E-02 | 0.3802724 |  | *IL20RA* | 4.77E-02 | -0.3439692 |
| 147 | *TRPM6* | 2.16E-03 | 0.3788911 |  | *MCTP1* | 1.51E-02 | -0.3416137 |
| 148 | *TXNDC5* | 1.29E-02 | 0.3786647 |  | *CLTCL1* | 1.17E-02 | -0.3409678 |
| 149 | *HDAC9* | 3.17E-02 | 0.3779714 |  | *KDM3B* | 9.64E-04 | -0.3404916 |
| 150 | *CDC42EP5* | 1.84E-02 | 0.3778303 |  | *ABCC3* | 1.88E-02 | -0.3403194 |
| 151 | *LDHA* | 3.15E-02 | 0.3773027 |  | *TFAP4* | 1.98E-02 | -0.338835 |
| 152 | *MDFI* | 1.42E-02 | 0.3766291 |  | *EPHA7* | 5.71E-03 | -0.338145 |
| 153 | *BTK* | 3.77E-02 | 0.3755941 |  | *GLG1* | 9.15E-04 | -0.3373841 |
| 154 | *ADAM8* | 2.38E-02 | 0.3753482 |  | *ZFP36L1* | 7.38E-03 | -0.3339275 |
| 155 | *NOD2* | 1.02E-02 | 0.375057 |  | *EHHADH* | 1.82E-02 | -0.333786 |
| 156 | *ENTPD6* | 3.74E-03 | 0.3742738 |  | *AP3B2* | 3.66E-02 | -0.3322474 |
| 157 | *KCNE3* | 2.57E-02 | 0.3702834 |  | *MSH2* | 2.55E-02 | -0.3295378 |
| 158 | *CYTIP* | 4.00E-02 | 0.3699573 |  | *RNF128* | 2.60E-02 | -0.3284846 |
| 159 | *TMPRSS2* | 4.74E-03 | 0.3692424 |  | *PANK1* | 3.00E-02 | -0.3283291 |
| 160 | *YRDC* | 1.04E-02 | 0.3683104 |  | *PTCH1* | 4.05E-02 | -0.3272582 |
| 161 | *FGL2* | 2.89E-02 | 0.3680995 |  | *PPP3CB* | 1.69E-03 | -0.3269364 |
| 162 | *RGS1* | 3.32E-02 | 0.3660157 |  | *SORBS2* | 1.49E-02 | -0.3268862 |
| 163 | *RIPK2* | 2.47E-02 | 0.3642661 |  | *ANKS1B* | 1.18E-02 | -0.3256939 |
| 164 | *CDC37* | 1.55E-03 | 0.3635374 |  | *PCDHB1* | 3.70E-02 | -0.3256853 |
| 165 | *SIGLEC10* | 4.04E-02 | 0.3626353 |  | *RHOBTB3* | 8.18E-03 | -0.3253452 |
| 166 | *S100P* | 4.74E-02 | 0.3605981 |  | *FKBP9* | 1.44E-02 | -0.3248177 |
| 167 | *USE1* | 2.33E-03 | 0.356854 |  | *PTPRF* | 9.64E-03 | -0.3229298 |
| 168 | *MAP3K8* | 1.22E-02 | 0.3543458 |  | *NHS* | 5.11E-03 | -0.3227428 |
| 169 | *PREB* | 7.11E-03 | 0.3529513 |  | *SMC1A* | 4.56E-03 | -0.321828 |
| 170 | *NEK6* | 2.34E-02 | 0.3528229 |  | *NID1* | 2.16E-02 | -0.3217982 |
| 171 | *PLGLB1* | 3.89E-02 | 0.3508222 |  | *SIK2* | 1.48E-03 | -0.3203447 |
| 172 | *SEC24D* | 1.86E-02 | 0.3480824 |  | *WDR7* | 2.32E-03 | -0.3203178 |
| 173 | *PPP1R3D* | 3.26E-03 | 0.3431114 |  | *SUMF1* | 5.75E-03 | -0.3199697 |
| 174 | *NFIL3* | 1.54E-02 | 0.3427793 |  | *KBTBD3* | 2.53E-02 | -0.3194948 |
| 175 | *EPS8L1* | 1.08E-02 | 0.3426904 |  | *CLSTN1* | 5.44E-03 | -0.3193144 |
| 176 | *LYSMD3* | 1.51E-02 | 0.3423727 |  | *SLC25A36* | 3.21E-02 | -0.3180662 |
| 177 | *SCIN* | 3.12E-02 | 0.3421214 |  | *SLAIN1* | 6.32E-03 | -0.3176843 |
| 178 | *ENDOD1* | 7.87E-03 | 0.3408862 |  | *SPRY1* | 1.19E-02 | -0.3170106 |
| 179 | *TMBIM1* | 1.41E-02 | 0.33989 |  | *MYLK3* | 1.69E-02 | -0.3150934 |
| 180 | *SAT2* | 7.33E-04 | 0.3396995 |  | *MANSC1* | 3.85E-02 | -0.3132726 |
| 181 | *ALG1L* | 2.62E-02 | 0.3396308 |  | *IP6K3* | 2.67E-02 | -0.3132327 |
| 182 | *RABAC1* | 1.81E-03 | 0.3380684 |  | *NUDT6* | 1.46E-02 | -0.3129566 |
| 183 | *AMN* | 3.34E-02 | 0.3379976 |  | *CRY2* | 3.20E-02 | -0.3115901 |
| 184 | *GCNT2* | 4.21E-03 | 0.3374265 |  | *CLCN4* | 5.51E-03 | -0.3111594 |
| 185 | *FAM83D* | 3.21E-02 | 0.3374138 |  | *GSTM3* | 4.50E-02 | -0.3097651 |
| 186 | *FAM110C* | 2.94E-02 | 0.3353064 |  | *PRKDC* | 6.64E-04 | -0.3097418 |
| 187 | *COX6B2* | 2.06E-02 | 0.3335644 |  | *CHST3* | 3.78E-02 | -0.3085334 |
| 188 | *GNE* | 2.77E-02 | 0.3324533 |  | *SLC25A12* | 1.40E-02 | -0.3075884 |
| 189 | *CHDH* | 2.73E-02 | 0.3316396 |  | *TDRD1* | 1.16E-02 | -0.3074394 |
| 190 | *KLHL5* | 1.07E-02 | 0.3309823 |  | *PCDHB4* | 3.03E-02 | -0.3060285 |
| 191 | *GNA15* | 5.93E-03 | 0.330868 |  | *PKD1L3* | 4.04E-02 | -0.3053117 |
| 192 | *ARHGAP10* | 4.89E-02 | 0.3307679 |  | *FKBP5* | 3.76E-02 | -0.3050855 |
| 193 | *DPYSL3* | 1.41E-02 | 0.3307306 |  | *NSUN7* | 3.20E-02 | -0.3046497 |
| 194 | *PLEKHN1* | 3.68E-02 | 0.3304875 |  | *ATXN1* | 5.83E-03 | -0.304336 |
| 195 | *P2RX4* | 1.89E-02 | 0.3284377 |  | *ERLIN2* | 3.51E-03 | -0.3042906 |
| 196 | *SULT1C2* | 3.28E-02 | 0.3282047 |  | *GEM* | 1.36E-02 | -0.3015652 |
| 197 | *ICA1* | 2.92E-03 | 0.3281418 |  | *LDOC1* | 1.47E-02 | -0.3006471 |
| 198 | *RAB12* | 1.12E-02 | 0.3263082 |  |  |  |  |
| 199 | *SLC39A8* | 3.63E-02 | 0.3261463 |  |  |  |  |
| 200 | *RGS10* | 2.82E-02 | 0.3261159 |  |  |  |  |
| 201 | *TMEM71* | 4.61E-03 | 0.3256459 |  |  |  |  |
| 202 | *CDH23* | 4.58E-02 | 0.3256055 |  |  |  |  |
| 203 | *SEC14L1* | 8.31E-03 | 0.324688 |  |  |  |  |
| 204 | *DGKD* | 3.37E-02 | 0.3242807 |  |  |  |  |
| 205 | *SDC2* | 1.36E-02 | 0.3242356 |  |  |  |  |
| 206 | *EIF4E3* | 1.38E-02 | 0.3226713 |  |  |  |  |
| 207 | *ZNF587* | 8.17E-03 | 0.3225755 |  |  |  |  |
| 208 | *OSTC* | 1.77E-02 | 0.3220474 |  |  |  |  |
| 209 | *PFKP* | 5.05E-03 | 0.3217802 |  |  |  |  |
| 210 | *IFIH1* | 4.47E-02 | 0.3186925 |  |  |  |  |
| 211 | *RELT* | 2.36E-02 | 0.3173007 |  |  |  |  |
| 212 | *GALNT7* | 2.85E-02 | 0.3171386 |  |  |  |  |
| 213 | *TIMP1* | 1.77E-02 | 0.3166849 |  |  |  |  |
| 214 | *SERTAD1* | 2.23E-02 | 0.3163673 |  |  |  |  |
| 215 | *USP15* | 1.23E-02 | 0.3149356 |  |  |  |  |
| 216 | *RGS19* | 2.78E-02 | 0.3144179 |  |  |  |  |
| 217 | *SEC11C* | 8.62E-03 | 0.3137219 |  |  |  |  |
| 218 | *ELOVL7* | 2.36E-02 | 0.3123429 |  |  |  |  |
| 219 | *ADM2* | 4.11E-02 | 0.3123259 |  |  |  |  |
| 220 | *STARD5* | 1.42E-02 | 0.3117769 |  |  |  |  |
| 221 | *ASPH* | 1.56E-02 | 0.3113795 |  |  |  |  |
| 222 | *PSMD13* | 5.74E-03 | 0.3113688 |  |  |  |  |
| 223 | *RANGRF* | 2.41E-02 | 0.3088172 |  |  |  |  |
| 224 | *PIP5K1A* | 1.63E-02 | 0.3086436 |  |  |  |  |
| 225 | *CDC42EP2* | 1.97E-02 | 0.3079681 |  |  |  |  |
| 226 | *LSP1* | 4.65E-02 | 0.3074594 |  |  |  |  |
| 227 | *BACE2* | 1.65E-02 | 0.3073248 |  |  |  |  |
| 228 | *MICA* | 1.47E-02 | 0.3071199 |  |  |  |  |
| 229 | *WSB2* | 1.02E-02 | 0.3069435 |  |  |  |  |
| 230 | *STXBP2* | 1.14E-02 | 0.3067189 |  |  |  |  |
| 231 | *ARFGAP3* | 5.97E-03 | 0.3051157 |  |  |  |  |
| 232 | *ILK* | 2.59E-03 | 0.3050874 |  |  |  |  |
| 233 | *ZFAND2A* | 4.34E-02 | 0.3047193 |  |  |  |  |
| 234 | *DISP1* | 2.44E-02 | 0.3030363 |  |  |  |  |
| 235 | *WDR45* | 8.19E-03 | 0.3013872 |  |  |  |  |
| 236 | *SDF2L1* | 3.78E-02 | 0.3010661 |  |  |  |  |
| 237 | *DPYD* | 2.14E-02 | 0.300403 |  |  |  |  |

^a^p-value, permutation *t*-test

^b^Fold change.

**Supplementary table 4. List of information used in immune cell signature**

| No. |  | *Monocyte* | *Dendritic cell* | *NK cell* | *T cell* | *B cell* | *Multiple* | *Lymphocyte* | *Neutriphil* |
| --- | --- | --- | --- | --- | --- | --- | --- | --- | --- |
| 1 |  | *AP1S2* | *GTP* | *CD244* | *ARH* | *ALG5* | *A2MP* | *ANP32E* | *ABTB1* |
| 2 |  | *BHLHB3* | *ABTB2* | *CHST12* | *ASPM* | *AMPD1* | *ACAT2* | *AP1GBP1* | *AMPD2* |
| 3 |  | *C21orf42* | *ADAM12* | *CLIC3* | *BCL11B* | *AP1* | *ACTR2* | *APOBEC3A* | *C5orf6* |
| 4 |  | *CASP1* | *ALOX15* | *IVNS1ABP* | *C21orf45* | *B4GALT3* | *ACTR3* | *ARL7* | *CCR3* |
| 5 |  | *CCL24* | *CBR3* | *KIR2DL1* | *CAMK4* | *BAFFR* | *AD023* | *ARPC5L* | *CDA* |
| 6 |  | *CCRL2* | *CCL13* | *KIR2DL2* | *CCDC5* | *BANK1* | *AD158* | *ASXL1* | *CKLFSF2* |
| 7 |  | *CD1D* | *CCL17* | *KIR2DL4* | *CCNA2* | *BCL11A* | *ADAM19* | *ATM* | *CLC* |
| 8 |  | *CDK5RAP2* | *CD1A* | *KIR2DS2* | *CCNB1* | *BCMA* | *ADAM8* | *BACH2* | *CREB5* |
| 9 |  | *CLECSF5* | *CD1B* | *KIR2DS5* | *CCNB2* | *BCNP1* | *ADCY7* | *BATF* | *CTBS* |
| 10 |  | *CSPG2* | *CD1E* | *KIR3DL1* | *CD3D* | *BLK* | *AGPS* | *BCL11B* | *DcR1* |
| 11 |  | *CTSD* | *CD86* | *KIR3DL2* | *CD3E* | *BLNK* | *AKAP13* | *BHLHB2* | *EST* |
| 12 |  | *CTSL* | *CG018* | *KIR3DS1* | *CD3G* | *BMP8B* | *AKNA* | *BTLA* | *FCGR2B* |
| 13 |  | *CXCL1* | *CHST7* | *KLRF1* | *CD5* | *BRDG1* | *ALG2* | *BTN3A2* | *FCGR3B* |
| 14 |  | *CXCL3* | *CLECSF13* | *NS1-BP* | *CD6* | *C14orf138* | *ALOX5* | *BTN3A3* | *FLJ10298* |
| 15 |  | *CXCL5* | *CLIC2* | *PTGDR* | *CD8A* | *C19orf10* | *ANKRD13* | *BUB3* | *FPRL1* |
| 16 |  | *CYBB* | *CNOT10* | *TGFBR1* | *CD8B1* | *C20orf31* | *ANP32A* | *C14orf61* | *FRAT2* |
| 17 |  | *DDEF1* | *CRLF2* | *YPEL1* | *CDC2* | *C21orf83B* | *AP1GBP1* | *C1orf33* | *GPR27* |
| 18 |  | *DFNA5* | *CXCL11* |  | *CDCA1* | *CD19* | *APBB1IP* | *C1QBP* | *GPR43* |
| 19 |  | *DTR* | *DCNP1* |  | *CDCA2* | *CD20* | *APOBEC3G* | *C21orf106* | *HSPA6* |
| 20 |  | *DUSP6* | *EHD4* |  | *CDCA5* | *CD79A* | *ARF6* | *C6orf129* | *IL8RA* |
| 21 |  | *EREG* | *EST* |  | *CPO* | *CD79B* | *ARHGAP15* | *CARD11* | *IL8RB* |
| 22 |  | *EST* | *FLJ13089* |  | *CTLA4* | *COPEB* | *ARHGAP25* | *CBLB* | *KIAA0779* |
| 23 |  | *FLJ20273* | *FLJ23230* |  | *CXCR6* | *CPNE5* | *ARHGAP9* | *CBX3* | *KIAA1126* |
| 24 |  | *FLJ20701* | *FLJ35936* |  | *DGKA* | *CXCR5* | *ARHH* | *CCND2* | *KRT23* |
| 25 |  | *GJB2* | *FLJ40614* |  | *DHX33* | *DDOST* | *ARL2* | *CD160* | *LENG4* |
| 26 |  | *GNA15* | *FLN29* |  | *DKFZp761K1824* | *DKFZp667L0210* | *ARNTL2* | *CD2* | *LENG5* |
| 27 |  | *GPR84* | *FNBP2* |  | *DNAJB1* | *DTNB* | *ARPC5* | *CD3Z* | *MAD* |
| 28 |  | *HS3ST3B1* | *FSCN1* |  | *DUFD1* | *EAF2* | *ARRB2* | *CD69* | *MGC10500* |
| 29 |  | *IER3* | *G1P3* |  | *DUSP2* | *EIF2AK3* | *ASF1B* | *CD7* | *MGC14126* |
| 30 |  | *IL19* | *GRSF1* |  | *EGLN3* | *ELL2* | *AXOT* | *CD96* | *MGC16353* |
| 31 |  | *IL1A* | *H6PD* |  | *EST* | *ERN1* | *BACH1* | *CDC25B* | *MPPE1* |
| 32 |  | *IL1B* | *HLA-DQ-alpha* |  | *FLJ11029* | *ERP70* | *BAL* | *CDK6* | *MSCP* |
| 33 |  | *IL1F9* | *HLA-DRB3* |  | *FLJ20249* | *EST* | *BAT4* | *CDT1* | *NCF4* |
| 34 |  | *IL1RN* | *HLA-DRB4* |  | *FTHFSDC1* | *FBXO18* | *BAZ1A* | *CENPH* | *NRBF-2* |
| 35 |  | *IL24* | *HMG20B* |  | *GPR15* | *FCRH1* | *BCL11A* | *CGI-09* | *PHC2* |
| 36 |  | *IL3RA* | *HMOX1* |  | *GZMK* | *FCRH2* | *BCL2* | *CHEK1* | *PROK2* |
| 37 |  | *IL6* | *HOZFP* |  | *H2AFX* | *FKBP11* | *BCL2L11* | *CHST12* | *RALB* |
| 38 |  | *INPP5B* | *IFIT1* |  | *HDGFRP3* | *FLJ20202* | *BCL6* | *CISH* | *RNF141* |
| 39 |  | *IPLA2* | *KIAA1404* |  | *HGFL* | *FLJ22625* | *BIC* | *CKS2* | *SEC14L1* |
| 40 |  | *IRAK2* | *Lectin* |  | *HMMR* | *FLJ23235* | *BIN2* | *CPSF5* | *SEPX1* |
| 41 |  | *LRRFIP1* | *LOC253782* |  | *HSPC150* | *FLJ31364* | *BIRC3* | *CRSP3* | *STX3A* |
| 42 |  | *MGC26226* | *LOC284018* |  | *ICOS* | *FLJ43842* | *BM-009* | *CTSW* | *TM4-B* |
| 43 |  | *MMP1* | *LOC93082* |  | *IL17F* | *FREB* | *BRAG* | *DDX21* | *VMP1* |
| 44 |  | *MMP14* | *MDA5* |  | *IL22* | *GNG7* | *BRD2* | *DEFA4* | *VNN2* |
| 45 |  | *MMP19* | *MDS010* |  | *IL9* | *GPRC5D* | *BRIP1* | *DKC1* | *XPO6* |
| 46 |  | *MMP9* | *MGC20700* |  | *INPP4B* | *HTPAP* | *BTN3A2* | *DKFZP564O0463* | |
| 47 |  | *MPEG1* | *MMP12* |  | *KIF14* | *IRTA2* | *BUB3* | *DKFZp762C1112* |  |
| 48 |  | *MS4A6A* | *MOB4A* |  | *LAG3* | *KIAA0102* | *C10orf9* | *DNAJC1* |  |
| 49 |  | *MTF1* | *MX1* |  | *LEF1* | *KIAA0125* | *C11orf21* | *DUT* |  |
| 50 |  | *NPC1* | *NAGPA* |  | *LEPROTL1* | *KIAA0746* | *C12orf14* | *EDG8* |  |
| 51 |  | *NRIP3* | *NR4A3* |  | *LRRN3* | *KIAA1384* | *C12orf5* | *EOMES* |  |
| 52 |  | *OK/KNS-CL.4* | *NT5C3* |  | *MELK* | *LOC220213* | *C13orf18* | *EPC1* |  |
| 53 |  | *PFKFB3* | *NUP62* |  | *MGC15763* | *LOC51061* | *C14orf106* | *EST* |  |
| 54 |  | *PLAUR* | *NYREN18* |  | *MGC3036* | *LZTFL1* | *C14orf118* | *EVER1* |  |
| 55 |  | *PLD1* | *OAS1* |  | *MGC5306* | *MAN1A1* | *C1D* | *EVER2* |  |
| 56 |  | *PLD3* | *OATPRP4* |  | *MKI67* | *MANEA* | *C1orf19* | *FAM29A* |  |
| 57 |  | *PPBP* | *PDL2* |  | *NDFIP2* | *MGC15606* | *C1orf22* | *FANCD2* |  |
| 58 |  | *PPIF* | *PLDN* |  | *NDNL2* | *MTND6* | *C1orf29* | *FBXO5* |  |
| 59 |  | *PRAM-1* | *PVRL2* |  | *PAICS* | *NALP7* | *C1orf33* | *FCRH3* |  |
| 60 |  | *PTX3* | *RAB30* |  | *PRC1* | *NGLY1* | *C1orf38* | *FEN1* |  |
| 61 |  | *RNU2* | *RAB9A* |  | *PRO2000* | *OSBPL10* | *C21orf91* | *FKSG14* |  |
| 62 |  | *RPEL1* | *RASSF4* |  | *PTP* | *PACAP* | *C2orf6* | *FKSG79* |  |
| 63 |  | *SERPINB2* | *RhoGAP2* |  | *RRM1* | *PAX5* | *C6orf187* | *FLJ12643* |  |
| 64 |  | *SERPINB7* | *SLC27A3* |  | *SDP35* | *PC4* | *C6orf32* | *FLJ12994* |  |
| 65 |  | *SERPINB9* | *SN* |  | *STK6* | *PNOC* | *C6orf67* | *FLJ20249* |  |
| 66 |  | *SGPP2* | *SPRED2* |  | *TCF7* | *POU2AF1* | *C6orf68* | *FLJ21438* |  |
| 67 |  | *SLC28A3* | *TBC1D13* |  | *TCRA* | *QRSL1* | *C6orf69* | *FLJ22457* |  |
| 68 |  | *SLC31A1* | *TTYH2* |  | *TOP2A* | *RALGPS2* | *C9orf19* | *FLJ32255* |  |
| 69 |  | *SLC3A2* | *UBD* |  | *TPX2* | *RPN1* | *CAMP* | *FLJ36837* |  |
| 70 |  | *SNX9* | *USP18* |  | *TRIM* | *SCFD1* | *cAMP-REBP* | *FLJ39739* |  |
| 71 |  | *SRC* |  |  | *TSBF1* | *SEC24A* | *CANX* | *FLJ39873* |  |
| 72 |  | *TCIRG1* |  |  | *UBE2C* | *SMCY* | *CAP1* | *FUS* |  |
| 73 |  | *TFPI2* |  |  | *UBE2S* | *SPATS2* | *CARP-1* | *GITR* |  |
| 74 |  | *TIEG* |  |  | *ZBED2* | *SPIB* | *CASP2* | *GLCCI1* |  |
| 75 |  | *TMEFF1* |  |  | *ZNRF1* | *SSR1* | *CASP3* | *GMPPB* |  |
| 76 |  | *TncRNA* |  |  | *ZWINT* | *STCH* | *CASP8* | *GNG2* |  |
| 77 |  | *VEG1* |  |  |  | *TCF3* | *CBL* | *GNLY* |  |
| 78 |  | *ZFHX1B* |  |  |  | *TCL1A* | *CBX5* | *GPR114* |  |
| 79 |  | *ZFYVE16* |  |  |  | *TLOC1* | *CCL1* | *GPR18* |  |
| 80 |  | *ZP3* |  |  |  | *TLR10* | *CCL20* | *GTPBP4* |  |
| 81 |  |  |  |  |  | *TRA1* | *CCL3* | *GZMA* |  |
| 82 |  |  |  |  |  | *TRAM1* | *CCL4* | *GZMB* |  |
| 83 |  |  |  |  |  | *TRAM2* | *CCL5* | *GZMH* |  |
| 84 |  |  |  |  |  | *TXNDC5* | *CCL8* | *GZMM* |  |
| 85 |  |  |  |  |  | *UBE2G1* | *CCR1* | *helios-3* |  |
| 86 |  |  |  |  |  | *UBE2J1* | *CCR2* | *HELLS* |  |
| 87 |  |  |  |  |  | *Ufm1* | *CCR5* | *HGLF* |  |
| 88 |  |  |  |  |  | *VIL2* | *CCR7* | *hIAN2* |  |
| 89 |  |  |  |  |  | *VPREB3* | *CCT2* | *HMGB1* |  |
| 90 |  |  |  |  |  | *WNT10A* | *CD164* | *IAN4L1* |  |
| 91 |  |  |  |  |  |  | *CD1C* | *ICAM2* |  |
| 92 |  |  |  |  |  |  | *CD37* | *IFI16* |  |
| 93 |  |  |  |  |  |  | *CD38* | *IFNG* |  |
| 94 |  |  |  |  |  |  | *CD44* | *IL2RB* |  |
| 95 |  |  |  |  |  |  | *CD48* | *ITGB7* |  |
| 96 |  |  |  |  |  |  | *CD53* | *ITK* |  |
| 97 |  |  |  |  |  |  | *CD58* | *ITM1* |  |
| 98 |  |  |  |  |  |  | *CD74* | *ITPKB* |  |
| 99 |  |  |  |  |  |  | *CD82* | *KA6* |  |
| 100 |  |  |  |  |  |  | *CD83* | *KIAA0101* |  |
| 101 |  |  |  |  |  |  | *CD84* | *KIAA0379* |  |
| 102 |  |  |  |  |  |  | *CD97* | *KIAA1509* |  |
| 103 |  |  |  |  |  |  | *CDC42* | *KIAA2010* |  |
| 104 |  |  |  |  |  |  | *CDC5L* | *KIF2C* |  |
| 105 |  |  |  |  |  |  | *CDK6* | *KIR2DL1* |  |
| 106 |  |  |  |  |  |  | *CDKN2D* | *KLF12* |  |
| 107 |  |  |  |  |  |  | *CDW52* | *KLIP1* |  |
| 108 |  |  |  |  |  |  | *CEB1* | *KLRB1* |  |
| 109 |  |  |  |  |  |  | *CECR1* | *KLRC3* |  |
| 110 |  |  |  |  |  |  | *CENTB1* | *KLRC4* |  |
| 111 |  |  |  |  |  |  | *CENTB2* | *KLRD1* |  |
| 112 |  |  |  |  |  |  | *CFLAR* | *KLRG1* |  |
| 113 |  |  |  |  |  |  | *CGI-09* | *KSP37* |  |
| 114 |  |  |  |  |  |  | *CGI-127* | *LAP2* |  |
| 115 |  |  |  |  |  |  | *CGI-37* | *LCK* |  |
| 116 |  |  |  |  |  |  | *CHC1L* | *LFIO9330* |  |
| 117 |  |  |  |  |  |  | *CHORDC1* | *LIF* |  |
| 118 |  |  |  |  |  |  | *CHS1* | *LOC197135* |  |
| 119 |  |  |  |  |  |  | *CHST11* | *LOC283989* |  |
| 120 |  |  |  |  |  |  | *CHST2* | *LOC342616* |  |
| 121 |  |  |  |  |  |  | *cig5* | *LOC348235* |  |
| 122 |  |  |  |  |  |  | *CKAP4* | *LOC51320* |  |
| 123 |  |  |  |  |  |  | *CKLF* | *LOC56854* |  |
| 124 |  |  |  |  |  |  | *CKLFSF8* | *LOC56902* |  |
| 125 |  |  |  |  |  |  | *CLECSF2* | *LTA* |  |
| 126 |  |  |  |  |  |  | *CLN2* | *LY9* |  |
| 127 |  |  |  |  |  |  | *CMRF-35H* | *LYAR* |  |
| 128 |  |  |  |  |  |  | *CNAP-1* | *MAD2L1* |  |
| 129 |  |  |  |  |  |  | *CNO* | *MAP4K1* |  |
| 130 |  |  |  |  |  |  | *CNOT6L* | *MCM10* |  |
| 131 |  |  |  |  |  |  | *CORO1A* | *MCM2* |  |
| 132 |  |  |  |  |  |  | *COTL1* | *MCM6* |  |
| 133 |  |  |  |  |  |  | *CR1L* | *MCM7* |  |
| 134 |  |  |  |  |  |  | *CRLF3* | *MCOLN1* |  |
| 135 |  |  |  |  |  |  | *CRTAM* | *MGAT2* |  |
| 136 |  |  |  |  |  |  | *CSF1* | *MGC16044* |  |
| 137 |  |  |  |  |  |  | *CSF2* | *MGC23244* |  |
| 138 |  |  |  |  |  |  | *CSF2RB* | *MGC24001* |  |
| 139 |  |  |  |  |  |  | *CSK* | *MGC3067* |  |
| 140 |  |  |  |  |  |  | *CSNK1G1* | *MGC5244* |  |
| 141 |  |  |  |  |  |  | *CST7* | *MGC61571* |  |
| 142 |  |  |  |  |  |  | *CTL1* | *MKI67IP* |  |
| 143 |  |  |  |  |  |  | *CTSC* | *MYBL1* |  |
| 144 |  |  |  |  |  |  | *CTSS* | *NCR3* |  |
| 145 |  |  |  |  |  |  | *CUGBP2* | *NDFIP2* |  |
| 146 |  |  |  |  |  |  | *CUL1* | *NFATC3* |  |
| 147 |  |  |  |  |  |  | *CX3CR1* | *NK4* |  |
| 148 |  |  |  |  |  |  | *CXCL10* | *NKG7* |  |
| 149 |  |  |  |  |  |  | *CXCL11* | *NOD3* |  |
| 150 |  |  |  |  |  |  | *CXCL9* | *NOP5/NOP58* |  |
| 151 |  |  |  |  |  |  | *CXCR4* | *NUSAP1* |  |
| 152 |  |  |  |  |  |  | *CXorf9* | *P2RX5* |  |
| 153 |  |  |  |  |  |  | *CYBA* | *PA2G4* |  |
| 154 |  |  |  |  |  |  | *CYLD* | *PAICS* |  |
| 155 |  |  |  |  |  |  | *CYSLTR1* | *PAI-RBP1* |  |
| 156 |  |  |  |  |  |  | *DAF* | *PAPOLA* |  |
| 157 |  |  |  |  |  |  | *DAPP1* | *PASK* |  |
| 158 |  |  |  |  |  |  | *DCAL1* | *PC4* |  |
| 159 |  |  |  |  |  |  | *DDHD1* | *PCNA* |  |
| 160 |  |  |  |  |  |  | *DDX17* | *PDK1* |  |
| 161 |  |  |  |  |  |  | *DDX39* | *PHF19* |  |
| 162 |  |  |  |  |  |  | *DEDD2* | *PIM2* |  |
| 163 |  |  |  |  |  |  | *DEF6* | *POLR1B* |  |
| 164 |  |  |  |  |  |  | *DEFA3* | *PPP1R2* |  |
| 165 |  |  |  |  |  |  | *DENR* | *PRDM2* |  |
| 166 |  |  |  |  |  |  | *DESR1* | *PRF1* |  |
| 167 |  |  |  |  |  |  | *DHX36* | *PRKCH* |  |
| 168 |  |  |  |  |  |  | *DKFZp313J1810* | *PRKCQ* |  |
| 169 |  |  |  |  |  |  | *DKFZP434B195* | *PRKX* |  |
| 170 |  |  |  |  |  |  | *DKFZp547A023* | *PRO2000* |  |
| 171 |  |  |  |  |  |  | *DKFZp547E052* | *PRSS1* |  |
| 172 |  |  |  |  |  |  | *DKFZP564J0863* | *PTGDR* |  |
| 173 |  |  |  |  |  |  | *DKFZp667L0210* | *PTPN4* |  |
| 174 |  |  |  |  |  |  | *DKFZp686E1929* | *PTPN7* |  |
| 175 |  |  |  |  |  |  | *DKFZP761E1824* | *PTPRCAP* |  |
| 176 |  |  |  |  |  |  | *DKFZp761P0423* | *PTTG1* |  |
| 177 |  |  |  |  |  |  | *DNAH3* | *RACGAP1* |  |
| 178 |  |  |  |  |  |  | *DNAJ* | *RAMP* |  |
| 179 |  |  |  |  |  |  | *DNAJC3* | *RASA2* |  |
| 180 |  |  |  |  |  |  | *DNCH1* | *RASGRP2* |  |
| 181 |  |  |  |  |  |  | *DNMT1* | *RFC4* |  |
| 182 |  |  |  |  |  |  | *DOCK10* | *RIOK1* |  |
| 183 |  |  |  |  |  |  | *DOCK2* | *RNF125* |  |
| 184 |  |  |  |  |  |  | *DOCK8* | *RORA* |  |
| 185 |  |  |  |  |  |  | *DRAK2* | *RPA3* |  |
| 186 |  |  |  |  |  |  | *DSIPI* | *RPIA* |  |
| 187 |  |  |  |  |  |  | *DUSP4* | *RRM2* |  |
| 188 |  |  |  |  |  |  | *DUSP5* | *SAMD3* |  |
| 189 |  |  |  |  |  |  | *DYRK2* | *SDF2L1* |  |
| 190 |  |  |  |  |  |  | *EAF1* | *44805* |  |
| 191 |  |  |  |  |  |  | *EBI2* | *44810* |  |
| 192 |  |  |  |  |  |  | *EDG6* | *SFRS2* |  |
| 193 |  |  |  |  |  |  | *EED* | *SH2D2A* |  |
| 194 |  |  |  |  |  |  | *EGLN1* | *SLC7A5* |  |
| 195 |  |  |  |  |  |  | *EIF2C2* | *SMC2L1* |  |
| 196 |  |  |  |  |  |  | *EIF2S3* | *SMC4L1* |  |
| 197 |  |  |  |  |  |  | *EIF5* | *SNRPA1* |  |
| 198 |  |  |  |  |  |  |  | *SOS1* |  |
| 199 |  |  |  |  |  |  |  | *SP100* |  |
| 200 |  |  |  |  |  |  |  | *SP4* |  |
| 201 |  |  |  |  |  |  |  | *SSR1* |  |
| 202 |  |  |  |  |  |  |  | *STAT4* |  |
| 203 |  |  |  |  |  |  |  | *STIP1* |  |
| 204 |  |  |  |  |  |  |  | *SYNE2* |  |
| 205 |  |  |  |  |  |  |  | *T2BP* |  |
| 206 |  |  |  |  |  |  |  | *TAF4B* |  |
| 207 |  |  |  |  |  |  |  | *TBX21* |  |
| 208 |  |  |  |  |  |  |  | *TCF19* |  |
| 209 |  |  |  |  |  |  |  | *TCF3* |  |
| 210 |  |  |  |  |  |  |  | *TNFRSF7* |  |
| 211 |  |  |  |  |  |  |  | *TNFSF6* |  |
| 212 |  |  |  |  |  |  |  | *TOP2A* |  |
| 213 |  |  |  |  |  |  |  | *TOSO* |  |
| 214 |  |  |  |  |  |  |  | *TRA2* |  |
| 215 |  |  |  |  |  |  |  | *TRIPIN* |  |
| 216 |  |  |  |  |  |  |  | *TYMS* |  |
| 217 |  |  |  |  |  |  |  | *UHRF1* |  |
| 218 |  |  |  |  |  |  |  | *UNC84B* |  |
| 219 |  |  |  |  |  |  |  | *VLIG-1* |  |
| 220 |  |  |  |  |  |  |  | *WBSCR22* |  |
| 221 |  |  |  |  |  |  |  | *WDR4* |  |
| 222 |  |  |  |  |  |  |  | *XCL1* |  |
| 223 |  |  |  |  |  |  |  | *XCL2* |  |
| 224 |  |  |  |  |  |  |  | *ZAP70* |  |
| 225 |  |  |  |  |  |  |  | *ZBP1* |  |
| 226 |  |  |  |  |  |  |  | *ZC3HAV1* |  |
| 227 |  |  |  |  |  |  |  | *ZNF101* |  |
| 228 |  |  |  |  |  |  |  | *ZNF137* |  |
| 229 |  |  |  |  |  |  |  | *ZNF367* |  |
| 230 |  |  |  |  |  |  |  | *ZNFN1A3* |  |
